# Parental knowledge, attitudes, and practices toward vaccinating their children against influenza: a cross-sectional study from China

**DOI:** 10.3389/fpubh.2024.1404506

**Published:** 2024-07-10

**Authors:** Shufeng He, Caijin Zhu, Xiaoyan Liu, Yanling Wang

**Affiliations:** Department of Infection Disease, Guangzhou Women and Children’s Medical Center, Guangzhou Medical University, Guangdong Provincial Clinical Research Center for Child Health, Guangzhou, China

**Keywords:** parents, influenza vaccination, knowledge, China, attitude

## Abstract

**Aims:**

Influenza infection is a health burden in children, and the influenza vaccine is an important prevention strategy for flu illness. Parents play a crucial role in children’s influenza vaccination. The study aimed to assess parental knowledge, attitudes, and practices (KAP) related to influenza illness for their children and explore factors that may impact their decisions.

**Methods:**

This cross-sectional study was conducted in a tertiary hospital in Guangzhou from November 2022 to April 2023. Answers to KAP questions regarding influenza illness and vaccination were summed, with a total KAP score of 20. Univariate and multivariate logistic regression models and linear regression models were conducted to explore the factors associated with influenza vaccination. The results were presented as odds ratios (ORs), *β*, and 95% confidence intervals (CIs).

**Results:**

Overall, 530 parents were respondents, of whom 162 (30.56%) had vaccinated their children during the past year. The mean KAP score (standard deviation) was 13.40 (3.57). Compared to parents who reported non-vaccinated for their children in the past year, the parents who reported an influenza vaccination have higher knowledge scores, attitude scores, practice scores, and total scores. Child body mass index, parental education level (under college), parental work (part-time), and more than two family members over 60 years old were negatively correlated with knowledge score. Child health condition and knowledge score were positively correlated with attitude score. Parental age was negatively associated with attitude score.

**Conclusion:**

Though high awareness about influenza illness and vaccination for parents, the coverage rate of influenza vaccination in children was lower in Guangzhou. Implementing public health policies is necessary to spread knowledge about influenza illness and vaccination and to promote the practice of receiving the influenza vaccine in children. Education campaigns would help change the attitudes of parents toward vaccinating their children against the flu.

## Introduction

Influenza is a common and serious infection marked by fever, cough, sore throat, chills, muscle ache, and general malaise (1, 2). It affects both adults and children, with children having a higher seasonal prevalence (20–30%) (3). Annually, influenza leads to up to 35,000 influenza-virus acute lower respiratory tract infection-associated deaths in children under 5 worldwide, posing a significant societal burden (4).

Annual influenza vaccination is the most effective preventive measure against the flu, especially in children, significantly reducing infection risk, severe complications, hospitalizations, and deaths (1, 5). Many countries, such as China, prioritize vaccinating children aged 6 months to 18 years (6). However, in China, vaccination coverage for children is low, approximately 11.9%, compared to higher rates in developed nations such as Europe and the United States (6–8).

Parents play a crucial role in deciding whether their children get vaccinated against influenza. Their understanding and attitudes toward vaccination are key influencers (9, 10). Misconceptions about vaccine safety and effectiveness, concerns about side effects, and insufficient awareness about vaccination’s importance contribute to parental vaccine hesitancy (11, 12). Additionally, cultural beliefs, socioeconomic status, healthcare access, and information sources shape parental vaccination decisions (13–15). This study aimed to explore the knowledge, attitudes, and practices (KAP) of parents regarding the influenza vaccine for their children and explore factors that may impact their decisions.

## Methods

### Study design, setting, and participants

From November 2022 to April 2023, parental caregivers of children aged 6 months to 18 years, who were treated at the department of infection disease of a tertiary hospital in Guangzhou, were selected as participants for this cross-sectional study. Exclusion criteria included (1) children aged <6 months or > 18 years old (2) missing information on influenza vaccination within the past year (3) individuals with allergies to the influenza vaccine or contraindication, and (4) individuals who were unable to complete the questionnaire due to writing or hearing disability.

At the department of infection disease in a selected tertiary hospital in Guangzhou, the investigator conducted face-to-face questionnaire surveys with parents in the waiting and observation rooms. All investigators were qualified and trained medical professionals, including physicians and nurses, who were required to work conscientiously. Sample size calculation was performed using the PASS 11.0 version, yielding an optimal size of 554, considering a 5% margin of error, a 95% confidence level, and a conservative estimate of vaccination coverage at 11.9% (7). Taking into account a shedding rate of 10%, a total sample size of 675 was needed for this survey. Finally, 619 parents participated in the study.

### Measurement tools

The survey included demographic information and influenza history for both children and parents. For children, socio-demographic details encompassed gender, age, height, weight, race, education level, health condition, and health insurance. Influenza history covered the children’s influenza history, that of their friends, their COVID-19 history, allergies to influenza vaccination, and contraindications for influenza vaccination. For parents, socio-demographic data included parental role, age, residence, marital status, education level, income, work, health condition, health insurance, and the presence of more than two family members over 60 years old in their household. Parents reported their influenza vaccination status. Influenza cases were diagnosed based on criteria established by the National Health Commission of the People’s Republic of China (16).

After reviewing relevant literature, a KAP questionnaire was developed and adapted to the local culture and context in Chinese (17, 18). The KAP questionnaire consists of 20 items derived from the literature review, with 7 items for Knowledge, 9 items for Attitude, and 4 items for Practice. Each correct response in the Knowledge section was assigned one point, while incorrect or “I do not know” responses received zero points. Participants were categorized based on their knowledge scores into poor knowledge (0–2), moderate knowledge (3–4), and high knowledge (5–7) groups. Attitude scores were based on varying response scales, including a 7-point disagree to agree scale, with one point awarded for each “Yes” response and zero points for “No” or “I do not know” answers. The Practice section included four statements related to influenza vaccination practices. Scores for the total questionnaire and each section ranged from 0 to 20, such as 0 to 7 for Knowledge, 0 to 9 for Attitude, and 0 to 4 for Practice. The Cronbach’s *α* coefficient for the KAP questionnaire was 0.71.

### Ethical consideration

The study was reviewed and approved by the Ethics Committees of Guangzhou Women and Children’s Medical Center, Guangzhou Medical University [No. 2022-257A01]. Written consent was obtained from all parents before filling out the questionnaires.

### Statistical analysis

Categorical variables were described using numbers and percentages, and a comparison between the two groups was made using the chi-square tests and Fisher exact tests. The normality of quantitative data was tested using skewness and kurtosis, and the equality of variances was tested using Levene’s tests. Continuous variables were presented as means and standard deviation. Statistical differences between the two groups were detected utilizing Student’s *t*-test and Satterthwaite’s *t*-test for homogeneity and heterogeneity quantitative data. Potential covariates were identified using univariate logistic regression models and univariate linear regression analysis. Subsequently, univariate and multivariate logistic regression models and linear regression models were conducted to explore the factors associated with influenza vaccination in children’s parents. The results were reported as odds ratios (ORs), *β*, and 95% confidence intervals (CIs). All statistical analyses were performed using R version 4.3.1 (2023–06–16 ucrt). A two-tailed *p*-value of <0.50 was considered statistically significant.

## Results

### Characteristics of parents and children

Out of 619 questionnaires distributed, 89 were excluded due to missing information on influenza vaccination within the past year (*n* = 69), and allergies or contraindications to the influenza vaccine (*n* = 20). The characteristics of children and parents are shown in Tables 1, 2, respectively. Among the 530 respondents, 368 (69.43%) did not administer influenza vaccination to their children. The mean age of vaccinated children was 4.77 (3.49) years old. Out of the 530 included parents, 426 (80.38%) were mothers. The average age of parents was 34.06 (5.31) years old. Parents possessing a college education level accounted for 283 (53.4%). Concerning those without influenza vaccination, children with influenza vaccination were younger on average, and so smaller, with lower weight, with lower education level, and with younger parents on average (all *p* < 0.05).

**Table 1:** Characteristics for children with and without influenza vaccination within the past year.

Variables	Total (*n* = 530)	Influenza vaccination		*P*
		Yes (*n* = 162)	No (*n* = 368)	
Child gender, *n* (%)				0.810^*^
Male	310 (58.49)	93 (57.41)	217 (58.97)	
Female	220 (41.51)	69 (42.59)	151 (41.03)	
Child age, years, Mean (±SD)	4.32 (±3.26)	3.32 (±2.38)	4.77 (±3.49)	<0.001^&^
Child height, cm, Mean (±SD)	101.57 (±24.26)	94.58 (±19.17)	104.64 (±25.61)	<0.001^&^
Child weight, kg, Mean (±SD)	18.73 (±11.44)	16.65 (±10.81)	19.65 (±11.61)	0.004^&^
Child BMI, kg/m^2^, Mean (±SD)	17.36 (±5.46)	18.12 (±6.83)	17.02 (±4.71)	0.063^&^
Child monocytangina, *n* (%)				0.255^*^
No	469 (88.49)	139 (85.8)	330 (89.67)	
Yes	61 (11.51)	23 (14.2)	38 (10.33)	
Child education level, *n* (%)				<0.001^#^
Junior high school	11 (2.08)	0 (0)	11 (2.99)	
Kindergarten	194 (36.6)	63 (38.89)	131 (35.6)	
No	217 (40.94)	82 (50.62)	135 (36.68)	
Primary school	106 (20)	16 (9.88)	90 (24.46)	
Senior high school	2 (0.38)	1 (0.62)	1 (0.27)	
Child health condition, *n* (%)				0.252^#^
General	130 (24.53)	43 (26.54)	87 (23.64)	
Good	384 (72.45)	117 (72.22)	267 (72.55)	
Poor	16 (3.02)	2 (1.23)	14 (3.8)	
Child health insurance, *n* (%)				0.345^*^
Basic medical insurance	246 (46.42)	76 (46.91)	170 (46.2)	
Commercial insurance	44 (8.3)	18 (11.11)	26 (7.07)	
No	46 (8.68)	11 (6.79)	35 (9.51)	
Rural cooperative medical insurance	194 (36.6)	57 (35.19)	137 (37.23)	
Child having influenza, *n* (%)				1.000^*^
No	439 (82.83)	134 (82.72)	305 (82.88)	
Yes	91 (17.17)	28 (17.28)	63 (17.12)	
Child friend having influenza, *n* (%)				0.192^*^
No	377 (71.13)	122 (75.31)	255 (69.29)	
Yes	153 (28.87)	40 (24.69)	113 (30.71)	
Child having COVID-19, *n* (%)				0.110^*^
No	328 (61.89)	109 (67.28)	219 (59.51)	
Yes	202 (38.11)	53 (32.72)	149 (40.49)	
Child friend having COVID-19, *n* (%)				0.080^*^
No	292 (55.09)	99 (61.11)	193 (52.45)	
Yes	238 (44.91)	63 (38.89)	175 (47.55)	

SD, standard deviation; ^&^Satterthwaite’s *t*-test; ^*^chi-square test; ^#^Fisher’s exact test.

**Table 2:** Characteristics for parents with and without influenza vaccination for their children within the past year.

Variables	Total (*n* = 530)	Influenza vaccination		*P*
		Yes (*n* = 162)	No (*n* = 368)	
Parents, *n* (%)				0.263^*^
Father	104 (19.62)	37 (22.84)	67 (18.21)	
Mother	426 (80.38)	125 (77.16)	301 (81.79)	
Parents age, years, Mean (±SD)	34.06 (±5.31)	33.28 (±5.35)	34.41 (±5.26)	0.023^&^
Residence, *n* (%)				0.176^*^
Town	370 (69.81)	106 (65.43)	264 (71.74)	
Village	160 (30.19)	56 (34.57)	104 (28.26)	
Marital status, *n* (%)				0.902^#^
Divorced	7 (1.32)	1 (0.62)	6 (1.63)	
Married	519 (97.92)	160 (98.77)	359 (97.55)	
Spinsterhood	1 (0.19)	0 (0)	1 (0.27)	
Widowed	3 (0.57)	1 (0.62)	2 (0.54)	
Education level, *n* (%)				0.848^#^
Postgraduate	32 (6.04)	7 (4.32)	25 (6.79)	
College	283 (53.4)	87 (53.7)	196 (53.26)	
Senior high school	98 (18.49)	31 (19.14)	67 (18.21)	
Junior high school	103 (19.43)	32 (19.75)	71 (19.29)	
Primary and below	14 (2.64)	5 (3.09)	9 (2.45)	
Income, *n* (%)				0.511^*^
>10 k	193 (36.42)	60 (37.04)	133 (36.14)	
0–5 k	179 (33.77)	59 (36.42)	120 (32.61)	
5–10 k	158 (29.81)	43 (26.54)	115 (31.25)	
Work, *n* (%)				0.126^*^
Full-time	289 (54.53)	84 (51.85)	205 (55.71)	
Others	106 (20)	33 (20.37)	73 (19.84)	
Part-time	97 (18.3)	27 (16.67)	70 (19.02)	
Unemployed	38 (7.17)	18 (11.11)	20 (5.43)	
Parents health condition, *n* (%)				0.244^#^
General	49 (9.25)	19 (11.73)	30 (8.15)	
Good	479 (90.38)	142 (87.65)	337 (91.58)	
Poor	2 (0.38)	1 (0.62)	1 (0.27)	
Parents health insurance, *n* (%)				0.589^*^
Basic medical insurance	334 (63.02)	102 (62.96)	232 (63.04)	
Commercial insurance	58 (10.94)	20 (12.35)	38 (10.33)	
No	23 (4.34)	9 (5.56)	14 (3.8)	
Rural cooperative medical insurance	115 (21.7)	31 (19.14)	84 (22.83)	
Parents influenza vaccination, *n* (%)				1.000^*^
No	508 (95.85)	155 (95.68)	353 (95.92)	
Yes	22 (4.15)	7 (4.32)	15 (4.08)	
Parents influenza vaccination time, *n* (%)				0.819^#^
0	508 (95.85)	155 (95.68)	353 (95.92)	
1	6 (1.13)	1 (0.62)	5 (1.36)	
2	4 (0.75)	2 (1.23)	2 (0.54)	
3	7 (1.32)	2 (1.23)	5 (1.36)	
4	5 (0.94)	2 (1.23)	3 (0.82)	
Parents having influenza, *n* (%)				0.330^*^
No	490 (92.45)	153 (94.44)	337 (91.58)	
Yes	40 (7.55)	9 (5.56)	31 (8.42)	
Parents friend having influenza, *n* (%)				0.087^*^
No	391 (73.77)	128 (79.01)	263 (71.47)	
Yes	139 (26.23)	34 (20.99)	105 (28.53)	
Parents having COVID-19, *n* (%)				0.352^*^
No	303 (57.17)	98 (60.49)	205 (55.71)	
Yes	227 (42.83)	64 (39.51)	163 (44.29)	
Parents friend having COVID-19, *n* (%)				0.080^*^
No	292 (55.09)	99 (61.11)	193 (52.45)	
Yes	238 (44.91)	63 (38.89)	175 (47.55)	
More than two family members over 60 years old, *n* (%)				0.520^*^
0	105 (19.81)	32 (19.75)	73 (19.84)	
1	115 (21.7)	40 (24.69)	75 (20.38)	
≥ 2	310 (58.49)	90 (55.56)	220 (59.78)	
Number of children, *n* (%)				0.224^*^
1	200 (37.74)	53 (32.72)	147 (39.95)	
2	256 (48.3)	87 (53.7)	169 (45.92)	
≥ 3	74 (13.96)	22 (13.58)	52 (14.13)	

SD, standard deviation; ^&^Student’s *t*-test; ^*^chi-square test; ^#^Fisher’s exact test.

### Knowledge, attitudes, and practices of parents regarding the influenza vaccine for their children

**Figure 1:**
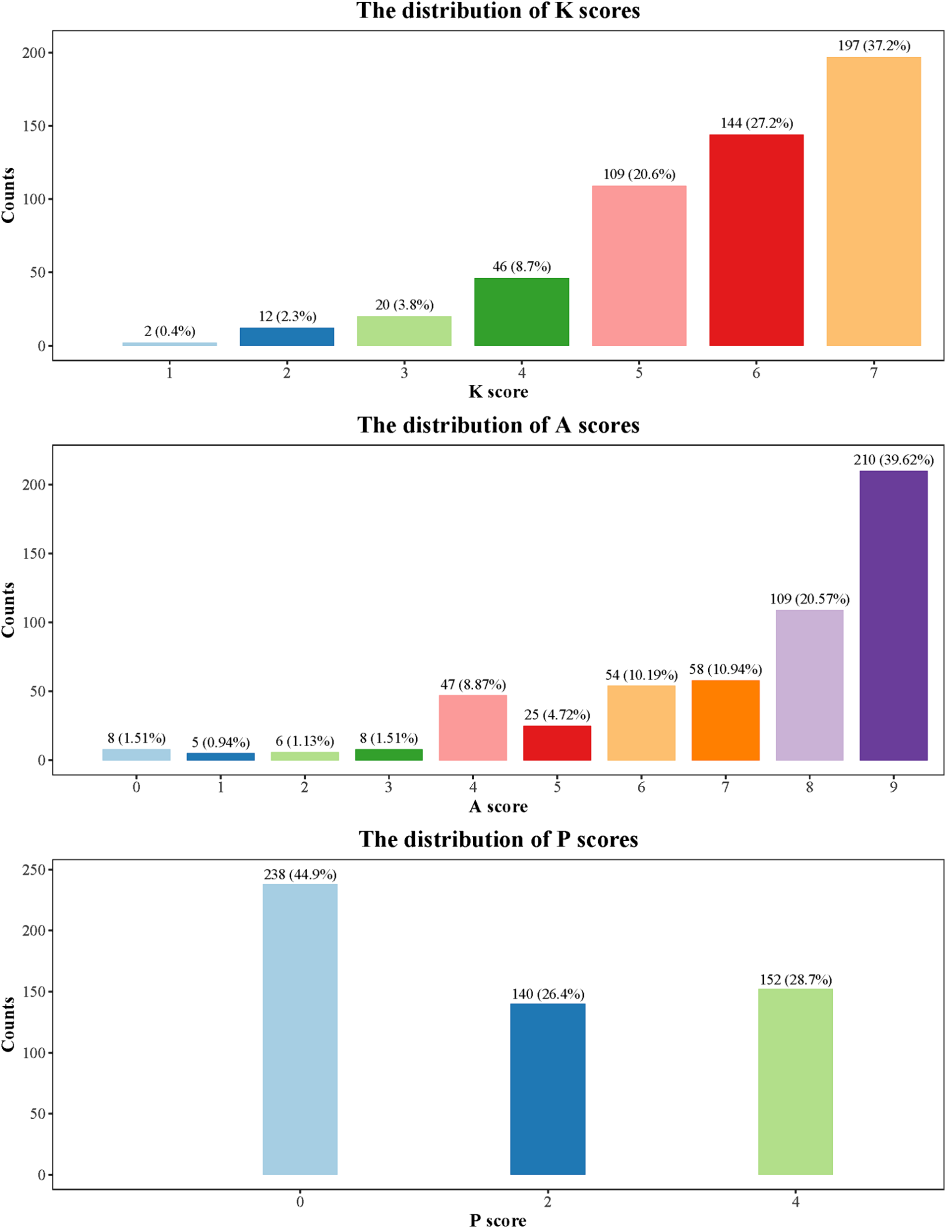
Distribution of KAP scores.

Table 3 shows the KAP scores for responses to influenza vaccination. The mean KAP score was 13.40 (3.57). The scores for knowledge, attitudes, and practices were 5.77 (1.29), 7.26 (2.10), and 1.68 (1.69), respectively. Compared to parents who reported without vaccinating their children against influenza in the past year, parents who reported an influenza vaccination have higher scores for knowledge, attitudes, practices, and total score (all *p* < 0.001). The distributions of scores for KAP are presented in Figure 1. Interestingly, despite reported high knowledge and attitude scores, the practice score remained low. Furthermore, 238 (44.9%) respondents have a practice score of 0.

**Table 3:** Scores of knowledge, attitude, and practice for parents.

Variables	Total (*n* = 530)	Influenza vaccination		*P*
		Yes (*n* = 162)	No (*n* = 368)	
K score, Mean (±SD)	5.77 (±1.29)	6.01 (±1.11)	5.67 (±1.35)	0.003^*^
A score, Mean (±SD)	7.26 (±2.10)	7.80 (±1.69)	7.02 (±2.22)	<0.001^*^
P score, Mean (±SD)	1.68 (±1.69)	3.88 (±0.48)	0.71 (±0.96)	<0.001^*^
Total score, Mean (±SD)	13.40 (±3.57)	16.33 (±2.38)	12.11 (±3.23)	<0.001^*^

SD, standard deviation; ^*^Satterthwaite’s *t*-test. K, knowledge; A, attitude; P, practice.

A total of 450 (84.91%) parents showed a high knowledge level toward influenza vaccination. The most correctly answered knowledge-related question was “Influenza can spread from person to person” with 506 (95.47%). Table 4 shows the questions of the knowledge section as well as the frequency of response for each question.

**Table 4:** Frequency of response for each question on knowledge reported by parents.

Item no.	Knowledge items	Yes, *n* (%)	No, *n* (%)	Unknown, *n* (%)
Question1	The flu and the cold are the same thing.	74 (13.96)	418 (78.87)	38 (7.17)
Question2	Everyone can get the flu, but children are more likely to get it.	487 (91.89)	27 (5.09)	16 (3.02)
Question3	Influenza can spread from person to person.	506 (95.47)	15 (2.83)	9 (1.7)
Question4	Influenza can cause severe illness or death.	401 (75.66)	47 (8.87)	82 (15.47)
Question5	You cannot catch the flu by touching surfaces or objects with the flu virus and then touching your own mouth, nose or eyes.	112 (21.13)	394 (74.34)	24 (4.53)
Question6	The best way to prevent the flu is to get a flu vaccine every year.	436 (82.26)	42 (7.92)	52 (9.81)
Question7	You can get a flu shot at any time, but it should be done by the end of October each year.	354 (66.79)	38 (7.17)	138 (26.04)

The questions about vaccine willingness and vaccine hesitancy are shown in Tables 5, 6, respectively. The table shows that 478 (90.19%) respondents regarded the vaccine as necessary, and 376 (70.94%) of respondents were planning to give their children the influenza vaccine. A total of 440 (83.02%) respondents were willing to pay the price of the influenza vaccine for their children, and 478 (90.19%) agreed to vaccinate their children if it was free of charge. In addition, 428 (80.75%) respondents thought it is safe to vaccinate children against influenza according to China’s standard immunization schedule. A total of 446 (84.15%) believed the flu vaccination was effective.

**Table 5:** Frequency of response for each question on vaccine willing reported by parents.

Item no.	Attitude items	Yes, *n* (%)	No, *n* (%)	Unknown, *n* (%)
Question8	Do you think it is necessary to have your child vaccinated against the flu?	478 (90.19)	19 (3.58)	33 (6.23)
Question9	If you could get the flu vaccine for free, would you give it to your child?	478 (90.19)	29 (5.47)	23 (4.34)
Question10	If you had to pay for the flu vaccine yourself, would you vaccinate your child?	440 (83.02)	44 (8.3)	46 (8.68)
Question11	Will you get your child the flu vaccine this year?	376 (70.94)	79 (14.91)	75 (14.15)

**Table 6:** Frequency of response for each question on vaccine hesitancy reported by parents.

Item no.	Attitudes items	Strongly disagree, *n* (%)	Medium disagree, *n* (%)	Slight disagree, *n* (%)	Unknown, *n* (%)	Slight agree, *n* (%)	Medium agree, *n* (%)	Strongly agree, *n* (%)
Question12	It is safe to vaccinate children against influenza according to China’s standard immunization schedule.	36 (6.79)	8 (1.51)	5 (0.94)	53 (10.00)	30 (5.66)	136 (25.66)	262 (49.43)
Question13	I believe the flu shot is effective.	39 (7.36)	11 (2.08)	2 (0.38)	32 (6.04)	50 (9.43)	161 (30.38)	235 (44.34)
Question14	If my child does not get the flu shot this year, I feel like he or she might get the flu.	49 (9.25)	20 (3.77)	6 (1.13)	131 (24.72)	76 (14.34)	108 (20.38)	140 (26.42)
Question15	How sure are you that you can follow your doctor’s advice and get your child vaccinated?	25 (4.72)	13 (2.45)	10 (1.89)	46 (8.68)	36 (6.79)	149 (28.11)	251 (47.36)
Question16	How sure are you that you can take the time to get your child vaccinated?	23 (4.34)	12 (2.26)	6 (1.13)	49 (9.25)	40 (7.55)	133 (25.09)	267 (50.38)

**Figure 2:**
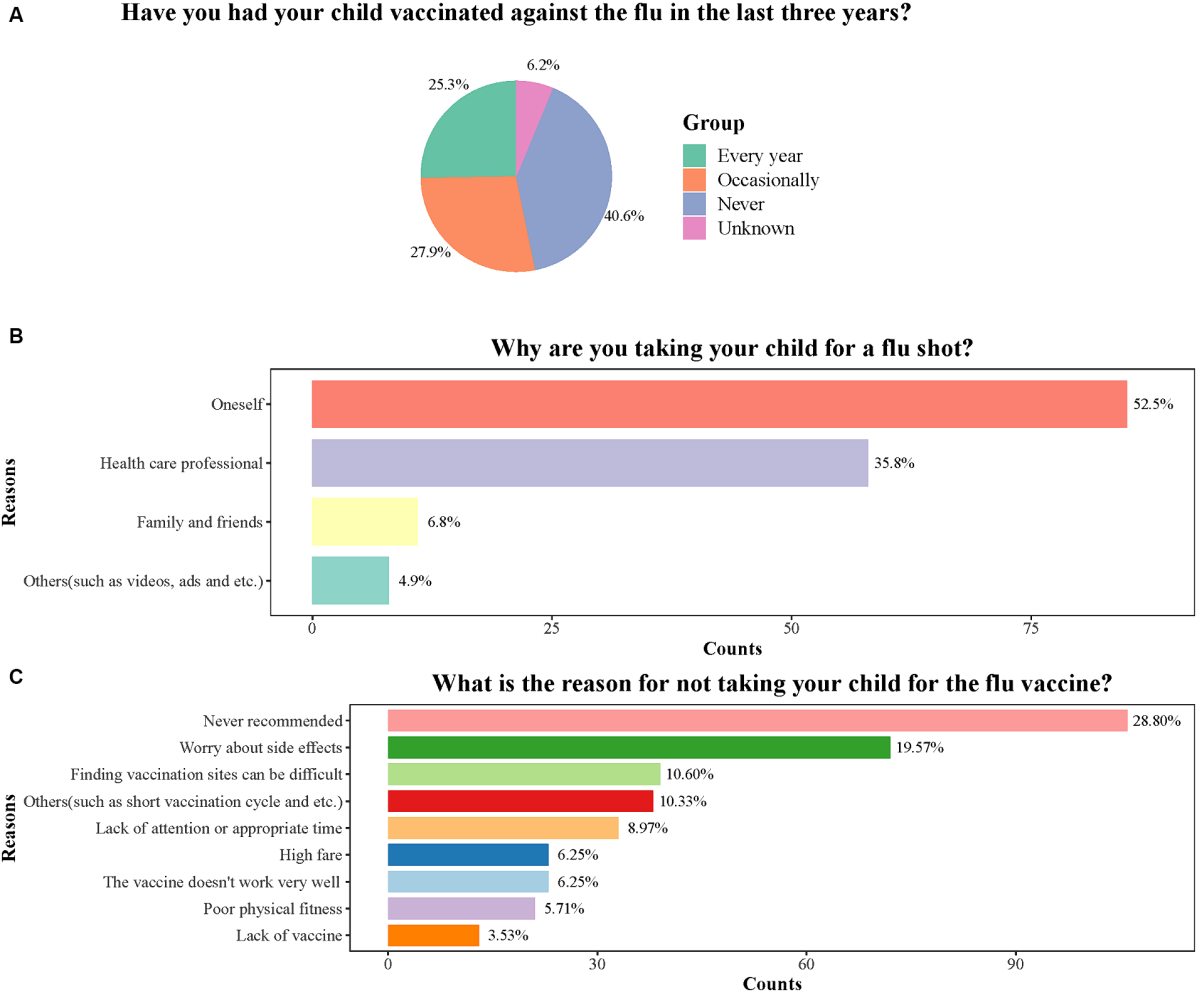
Distribution of four items in practice score. **(A)** The distribution of answers to the question “Have you had your child vaccinated against the flu in the last three years”. **(B)** The distribution of answers to the question “Why are you taking your child for a flu shot”. **(C)** The distribution of answers to the question “What is the reason for not taking your child for the flu vaccine”.

In total, 162 (30.56%) respondents reported vaccinating their children against influenza in the past year. The data distribution on four items in practices is presented in Figure 2. Among the respondents, 106 (27.9%) vaccinated their children against influenza occasionally, 134 (25.3%) vaccinated their children every year, and 215 (40.6%) never had their children vaccinated. Parents who got their children vaccinated against influenza were mainly driven by their decisions. Parents who did not administer the influenza vaccine to their children stated lack of recommendation by health providers as the most common reason (28.80%).

### Factors associated with children receiving influenza vaccination

In Supplementary Table S1, a high attitude score was associated with higher odds of children receiving the influenza vaccine (OR = 1.17, 95%CI: 1.05 to 1.31). Knowledge score was marginal significance with odds of children receiving the influenza vaccine (OR = 1.19, 95%CI: 1.00 to 1.43). Child body mass index (BMI) (*β* = −0.02, 95%CI: −0.04 to −0.01), parental education level (under college) (*β* = −1.34, 95%CI: −1.98 to −0.71), parental with part-time work (*β* = −0.29, 95%CI: −0.51 to −0.07), and more than two family members over 60 years old (*β* = −0.28, 95%CI: −0.49 to −0.07) were negatively correlated with knowledge score. Child health condition (*β* = 0.28, 95%CI: 0.10 to 0.47) and knowledge score (*β* = 0.26, 95%CI: 0.20 to 0.33) have a positive correlation with attitude score. Parental age (*β* = −0.02, 95%CI: −0.04 to −0.01) was negatively associated with attitude score, indicating that the younger the parents, the higher the score in vaccine attitudes. Influencing factors on knowledge score and attitude score are presented in Tables 7, 8, respectively.

**Table 7:** Influencing factors on knowledge score reported by parents.

	Model 1		Model 2	
Variables	β (95% CI)	*P*	β (95% CI)	*P*
Child sex				
Male	Ref			
Female	−0.03 (−0.21–0.14)	0.715		
Child age	−0.02 (−0.04–0.01)	0.255		
Child BMI	−0.02 (−0.04--0.01)	0.005	−0.02 (−0.04--0.01)	0.006
Child monocytangina				
No	Ref			
Yes	0.13 (−0.14–0.40)	0.342		
Child education				
No	Ref			
Kindergarten	0.15 (−0.05–0.34)	0.136		
Primary school	−0.07 (−0.30–0.16)	0.550		
Junior high school	−0.29 (−0.89–0.32)	0.354		
Senior high school	−1.34 (−2.73–0.05)	0.059		
Child health condition				
General	Ref			
Good	0.01 (−0.19–0.21)	0.944		
Poor	0.04 (−0.48–0.56)	0.882		
Child health insurance				
Basic medical insurance	Ref		Ref	
Commercial insurance	0.22 (−0.10–0.54)	0.181	0.03 (−0.32–0.38)	0.882
No	−0.16 (−0.47–0.16)	0.322	0.06 (−0.26–0.38)	0.719
Rural cooperative medical insurance	−0.24 (−0.43--0.05)	0.012	−0.04 (−0.26–0.18)	0.727
Child influenza				
No	Ref			
Yes	0.15 (−0.07–0.38)	0.183		
Child friend influenza				
No	Ref		Ref	
Yes	0.24 (0.05–0.42)	0.014	0.14 (−0.04–0.32)	0.127
Child having COVID-19				
No	Ref			
Yes	−0.10 (−0.27–0.08)	0.284		
Child friend having COVID-19				
No	Ref			
Yes	−0.02 (−0.20–0.15)	0.776		
Parents				
Father	Ref			
Mother	0.05 (−0.17–0.26)	0.669		
Parents age	−0.00 (−0.02–0.01)	0.699		
Parents residence				
Town	Ref		Ref	
Village	−0.33 (−0.51--0.14)	0.001	−0.02 (−0.22–0.19)	0.858
Parents marital status				
Divorced	Ref			
Married	0.27 (−0.48–1.01)	0.485		
Spinsterhood	−0.33 (−2.43–1.77)	0.757		
Widowed	0.70 (−0.66–2.06)	0.312		
Parental education level				
Postgraduate	Ref		Ref	
College	−0.29 (−0.64–0.05)	0.098	−0.32 (−0.67–0.04)	0.080
Senior high school	−0.70 (−1.08--0.32)	<0.001	−0.67 (−1.08--0.27)	0.001
Junior high school	−1.01 (−1.39--0.64)	<0.001	−1.02 (−1.46--0.58)	<0.001
Primary and below	−1.28 (−1.87--0.68)	<0.001	−1.34 (−1.98--0.71)	<0.001
Parents income				
>10 k	Ref		Ref	
0–5 k	−0.48 (−0.68--0.28)	<0.001	−0.07 (−0.31–0.16)	0.530
5–10 k	−0.12 (−0.33–0.08)	0.243	0.07 (−0.14–0.28)	0.528
Parents work				
Full-time	Ref		Ref	
Others	−0.16 (−0.38–0.07)	0.166	0.26 (0.03–0.50)	0.026
Part-time	−0.35 (−0.57--0.12)	0.003	−0.29 (−0.51--0.07)	0.009
Unemployed	−0.20 (−0.53–0.14)	0.256	0.18 (−0.15–0.51)	0.292
Parents health condition				
General	Ref			
Good	0.24 (−0.06–0.53)	0.115		
Poor	0.78 (−0.63–2.20)	0.279		
Parents health insurance				
Basic medical insurance	Ref		Ref	
Commercial insurance	0.35 (0.07–0.62)	0.014	0.24 (−0.06–0.55)	0.121
No	−0.14 (−0.55–0.28)	0.518	−0.02 (−0.44–0.41)	0.932
Rural cooperative medical insurance	−0.36 (−0.57--0.15)	0.001	0.01 (−0.25–0.27)	0.920
Parents influenza vaccination				
No	Ref			
Yes	0.22 (−0.21–0.65)	0.307		
Parents influenza vaccination time				
0	Ref			
1	0.70 (−0.10–1.51)	0.086		
2	0.38 (−0.60–1.36)	0.447		
3	−0.59 (−1.33–0.16)	0.123		
4	0.65 (−0.23–1.53)	0.146		
Parents having influenza				
No	Ref			
Yes	0.07 (−0.26–0.39)	0.684		
Parents friend having influenza				
No	Ref			
Yes	0.11 (−0.09–0.30)	0.285		
Parents having COVID-19				
No	Ref			
Yes	−0.14 (−0.31–0.03)	0.107		
Parents friend having COVID-19				
No	Ref			
Yes	−0.08 (−0.25–0.09)	0.372		
More than two family members over 60 years old				
0	Ref		Ref	
1	−0.04 (−0.30–0.22)	0.756	−0.03 (−0.28–0.22)	0.794
≥ 2	−0.31 (−0.53--0.09)	0.005	−0.28 (−0.49--0.07)	0.009
Number of children				
1	Ref		Ref	
2	−0.12 (−0.31–0.06)	0.192	0.09 (−0.09–0.26)	0.350
≥ 3	−0.49 (−0.76--0.23)	<0.001	−0.16 (−0.43–0.10)	0.214

*β*, coefficient; CI, confidence intervals; Ref, reference. BMI, body mass index. Model 1, Crude model. Model 2, Included variables with statistical differences in model 1.

**Table 8:** Influencing factors on attitude score reported by parents.

	Model 1		Model 2	
Variables	*β* (95% CI)	*P*	*β* (95% CI)	*P*
Child sex				
Male	Ref			
Female	0.04 (−0.13–0.21)	0.659		
Child age	−0.03 (−0.06−−0.01)	0.018	–0.01 (−0.04–0.02)	0.465
Child BMI	0.00 (−0.01–0.02)	0.865		
Child monocytangina				
No	Ref			
Yes	0.05 (−0.22–0.31)	0.730		
Child education				
No	Ref			
Kindergarten	0.08 (−0.11–0.27)	0.412		
Primary school	−0.23 (−0.46–0.00)	0.052		
Junior high school	−0.32 (−0.92–0.29)	0.303		
Senior high school	0.09 (−1.30–1.48)	0.895		
Child health condition				
General	Ref		Ref	
Good	0.24 (0.05–0.44)	0.015	0.28 (0.10–0.47)	0.003
Poor	−0.37 (−0.89–0.14)	0.157	−0.31 (−0.79–0.16)	0.197
Child health insurance				
Basic medical insurance	Ref			
Commercial insurance	0.17 (−0.15–0.49)	0.302		
No	−0.29 (−0.60–0.03)	0.073		
Rural cooperative medical insurance	0.17 (−0.01–0.36)	0.070		
Child having influenza				
No	Ref			
Yes	0.12 (−0.11–0.34)	0.308		
Child friend having influenza				
No	Ref		Ref	
Yes	0.24 (0.06–0.43)	0.011	0.16 (−0.02–0.34)	0.076
Child having COVID-19				
No	Ref			
Yes	0.11 (−0.06–0.29)	0.200		
Child friend having COVID-19				
No	Ref		Ref	
Yes	0.20 (0.03–0.37)	0.022	−0.27 (−0.69–0.16)	0.221
Parents				
Father	Ref			
Mother	0.00 (−0.21–0.22)	0.972		
Parental age	−0.03 (−0.04--0.01)	0.001	−0.02 (−0.04--0.01)	0.010
Parents residence				
Town	Ref			
Village	0.15 (−0.04–0.33)	0.116		
Parents marital status				
Divorced	Ref			
Married	0.33 (−0.42–1.08)	0.384		
Spinsterhood	−0.27 (−2.38–1.83)	0.800		
Widowed	0.20 (−1.15–1.56)	0.768		
Parents education				
Postgraduate	Ref			
College	0.09 (−0.27–0.46)	0.618		
Senior high school	−0.11 (−0.51–0.29)	0.575		
Junior high school	−0.08 (−0.48–0.31)	0.685		
Primary and below	−0.36 (−0.98–0.27)	0.265		
Parents income				
>10 k	Ref			
0–5 k	−0.04 (−0.24–0.16)	0.696		
5–10 k	0.14 (−0.07–0.35)	0.189		
Parents work				
Full-time	Ref			
Others	0.03 (−0.19–0.26)	0.768		
Part-time	−0.03 (−0.26–0.20)	0.797		
Unemployed	−0.04 (−0.38–0.30)	0.836		
Parents health condition				
General	Ref			
Good	0.24 (−0.05–0.54)	0.107		
Poor	0.10 (−1.32–1.51)	0.893		
Parents health insurance				
Basic medical insurance	Ref			
Commercial insurance	−0.05 (−0.33–0.23)	0.746		
No	−0.29 (−0.72–0.13)	0.174		
Rural cooperative medical insurance	0.05 (−0.16–0.27)	0.619		
Parents influenza vaccination				
No	Ref			
Yes	0.19 (−0.24–0.62)	0.388		
Parents influenza vaccination time				
0	Ref			
1	0.28 (−0.53–1.09)	0.494		
2	0.24 (−0.75–1.23)	0.630		
3	−0.05 (−0.80–0.70)	0.904		
4	0.36 (−0.52–1.25)	0.423		
Parents having influenza				
No	Ref			
Yes	0.10 (−0.22–0.42)	0.546		
Parents friend having influenza				
No	Ref			
Yes	0.17 (−0.02–0.37)	0.080		
Parents having COVID-19				
No	Ref		Ref	
Yes	0.18 (0.01–0.35)	0.038	0.10 (−0.19–0.38)	0.497
Parents friend having COVID-19				
No	Ref		Ref	
Yes	0.24 (0.06–0.41)	0.007	0.40 (−0.03–0.83)	0.067
More than two family members over 60 years old				
0	Ref			
1	0.17 (−0.09–0.44)	0.200		
≥ 2	−0.03 (−0.26–0.19)	0.766		
Number of children				
1	Ref			
2	0.03 (−0.15–0.22)	0.726		
≥ 3	−0.23 (−0.49–0.04)	0.094		
K score	0.27 (0.20–0.33)	<0.001	0.26 (0.20–0.33)	<0.001

*β*, coefficient; CI, confidence intervals; Ref, reference. BMI, body mass index. Model 1, Crude model. Model 2, Included variables with statistical differences in model 1.

## Discussion

Our study found most parents had a high knowledge level and positive attitudes toward influenza vaccination, while only 30.56% of parents reported vaccinating their children against influenza in the past year. The results indicated a gap between parents’ intention and their actual vaccination behaviors in the realm of healthcare practices. The primary reason cited for not vaccinating was the absence of a recommendation.

Parental knowledge significantly influences the intentions regarding children’s flu vaccination, as supported by Zhao et al. (19). A survey conducted in the Nanhai district of China showed a positive association between heightened levels of influenza knowledge and previous influenza information with a willingness to vaccinate (11).

Our study found that children’s BMI, parental education level, parental with part-time work, and having more than two family members over 60 years old were negatively related to knowledge score. Individuals with higher BMI often face social stigma and discrimination, potentially resulting in diminished health literacy and participation in preventative health practices (20). Therefore, these parents might have limited vaccination knowledge, leading to lower knowledge scores. Parental education level has been linked to decreased knowledge regarding vaccinations (21). Parents with lower education levels may have limited access to credible health information sources, impeding their ability to obtain accurate knowledge on influenza vaccination for their children. Moreover, parental part-time work and having two or more people over 60 years old in the household may contribute to lower knowledge scores due to time constraints and competing responsibilities. The presence of older adult family members may divert parents’ focus and time away from seeking vaccination information. Given the limited knowledge about influenza vaccination among Chinese parents, it is crucial for the China Centers for Disease Control and Prevention to offer comprehensive and accessible information to address this gap.

The majority of parents had favorable opinions toward the influenza vaccine. Parents showed high willingness (70.94%) to vaccinate their children against influenza in our study, indicating that China has done quite well in the publicity and implementation of the law on the prevention and control of infectious diseases (15). Moreover, our study found positive correlations of knowledge score, the health status of children with attitude score. Parental knowledge plays a crucial role in vaccination decision-making (22). Parents who possess accurate knowledge are more inclined to comprehend the advantages of influenza vaccination and make well-informed decisions for their children (10). Parents with children in good health may have had positive experiences with previous vaccinations, which influence their perspectives and beliefs regarding the effectiveness and significance of vaccines (23). A negative relationship was reported between parental age and knowledge score, as supported by Alenazi (22). Younger parents demonstrated higher scores in attitude categories due to their increased internet exposure, which facilitated access to online influenza-related information. Moreover, our findings indicated that as knowledge improved, attitudes became increasingly optimistic (24–26).

Only 30.56% of parents reported influenza vaccination for their children in the past year, which is lower compared to rates in various nations such as the U.S. (27), United Kingdom (28), Italy (29), and Saudi Arabia (22). The recommendations provided by physicians were crucial in promoting influenza vaccination among children (30, 31). A survey conducted in the U.S. showed that healthcare provider recommendations significantly influenced the uptake of influenza vaccine in children aged 9–13 years (32). Consequently, stakeholders should educate healthcare professionals about the guidelines for influenza immunization and enhance their knowledge on the subject. The commonly cited reason (28.80%) for parents not taking their children for influenza vaccination was the absence of recommendations, indicating the necessity to promote influenza vaccination. Media can be utilized more effectively to spread information about influenza illness and vaccination as it is an effective tool in increasing vaccination rates in children (33). Additionally, parents’ apprehension regarding potential side effects of the vaccine presented a barrier to childhood influenza vaccination, with various studies identifying concerns about vaccine safety as the primary reason for refusing vaccination (34, 35).

The status of influenza vaccination in children is suboptimal, indicating that healthcare providers should focus on improving the situation. Strategies should not only involve providing information but also address parent’s attitudes and concerns. Targeted intervention measures should be implemented based on influencing factors and individual characteristics of health education. Moreover, healthcare providers, serving as a vital and trustworthy source of vaccine-related information for the public, could benefit from additional training in professional ethics and specialized knowledge to enhance parental awareness (36, 37).

Several limitations should be recognized in the study. First, the study used a cross-sectional design with respondents sampled solely from one hospital. Future studies should encompass larger and multi-center samples. Second, health conditions for parents and children were obtained by self-reported, leading to response bias. Finally, the majority of parents were women (80.38%), raising concerns about gender bias. The extent to which decision-making regarding children’s influenza vaccination may vary between fathers and mothers remains uncertain.

## Conclusion

While there is increased awareness among parents about influenza vaccination, only 30.56% of them reported vaccinating their children against influenza in the past year. This underscores the need for interventions to boost vaccination rates, addressing not only knowledge and attitudes but also practical barriers that hinder parents from vaccinating their children. Strategies could involve targeted educational initiatives, enhanced access to vaccination services, and assistance for parents in scheduling and attending vaccination appointments. Encouraging physician recommendations for the influenza vaccine is vital and should be promoted in healthcare centers, clinics, and hospitals.

## Data availability statement

The raw data supporting the conclusions of this article will be made available by the authors, without undue reservation.

## Ethics statement

The studies involving humans were approved by Guangzhou Women and Children’s Medical Center, Guangzhou Medical University. The studies were conducted in accordance with the local legislation and institutional requirements. Written informed consent for participation in this study was provided by the participants’ legal guardians/next of kin.

## Author contributions

SH: Data curation, Formal analysis, Investigation, Methodology, Writing – review & editing. CZ: Data curation, Formal analysis, Investigation, Methodology, Writing – review & editing. XL: Data curation, Formal analysis, Investigation, Methodology, Writing – review & editing. YW: Conceptualization, Project administration, Supervision, Writing – review & editing.
