# Supplementary material for: Parental knowledge, attitudes, and practices toward vaccinating their children against influenza: a cross-sectional study from China
Source: Front Public Health. 2024 Jul 10;12:1404506. doi: 10.3389/fpubh.2024.1404506 (PMC11267369; doi:10.3389/fpubh.2024.1404506)
Supplement: Supplementary file 1 [file Table_1.DOCX]

Table S1 Influencing factors on influenza vaccination during the past year

|  | Model l |  | Model 2 |  |
| --- | --- | --- | --- | --- |
| Variables | OR (95% CI) | *P* | OR (95% CI) | *P* |
| Child sex |  |  |  |  |
| Male | Ref |  |  |  |
| Female | 1.07 (0.73-1.55) | 0.737 |  |  |
| Child age | 0.85 (0.80-0.91) | <0.001 | 0.89 (0.75-1.05) | 0.183 |
| Child BMI | 1.04 (1.00-1.07) | 0.035 | 1.03 (1.00-1.07) | 0.081 |
| Child monocytangina |  |  |  |  |
| No | Ref |  |  |  |
| Yes | 1.44 (0.83-2.50) | 0.200 |  |  |
| Child education level |  |  |  |  |
| No | Ref |  | Ref |  |
| Kindergarten | 0.79 (0.53-1.19) | 0.261 | 1.11 (0.60-2.06) | 0.750 |
| Primary school | 0.29 (0.16-0.53) | <0.001 | 0.69 (0.19-2.55) | 0.582 |
| Junior high school | 0.00 (0.00-Inf) | 0.982 | 0.00 (0.00-Inf) | 0.983 |
| Senior high school | 1.65 (0.10-26.68) | 0.726 | 9.44 (0.21-435.32) | 0.217 |
| Child health condition |  |  |  |  |
| General | Ref |  |  |  |
| Good | 0.89 (0.58-1.36) | 0.579 |  |  |
| Poor | 0.29 (0.06-1.33) | 0.111 |  |  |
| Child health insurance |  |  |  |  |
| Basic medical insurance | Ref |  |  |  |
| Commercial insurance | 1.55 (0.80-2.99) | 0.193 |  |  |
| No | 0.70 (0.34-1.46) | 0.344 |  |  |
| Rural cooperative medical insurance | 0.93 (0.62-1.40) | 0.732 |  |  |
| Child influenza |  |  |  |  |
| No | Ref |  |  |  |
| Yes | 1.01 (0.62-1.65) | 0.963 |  |  |
| Child friend influenza |  |  |  |  |
| No | Ref |  |  |  |
| Yes | 0.74 (0.49-1.13) | 0.160 |  |  |
| Child COVID-19 |  |  |  |  |
| No | Ref |  |  |  |
| Yes | 0.71 (0.48-1.05) | 0.090 |  |  |
| Child friend COVID-19 |  |  |  |  |
| No | Ref |  |  |  |
| Yes | 0.70 (0.48-1.02) | 0.065 |  |  |
| Parents |  |  |  |  |
| Father | Ref |  |  |  |
| Mother | 0.75 (0.48-1.18) | 0.217 |  |  |
| Parents age | 0.96 (0.93-0.99) | 0.024 | 1.01 (0.96-1.05) | 0.773 |
| Parents residence |  |  |  |  |
| Town | Ref |  |  |  |
| Village | 1.34 (0.90-1.99) | 0.146 |  |  |
| Parents marital status |  |  |  |  |
| Divorced | Ref |  |  |  |
| Married | 2.67 (0.32-22.39) | 0.364 |  |  |
| Spinsterhood | 0.00 (0.00-Inf) | 0.982 |  |  |
| Widowed | 3.00 (0.12-73.65) | 0.501 |  |  |
| Parents education level |  |  |  |  |
| Postgraduate | Ref |  |  |  |
| College | 1.59 (0.66-3.80) | 0.302 |  |  |
| Senior high school | 1.65 (0.65-4.23) | 0.295 |  |  |
| Junior high school | 1.61 (0.63-4.11) | 0.319 |  |  |
| Primary and below | 1.98 (0.50-7.87) | 0.330 |  |  |
| Parents income |  |  |  |  |
| >10 k | Ref |  |  |  |
| 0-5 k | 1.09 (0.70-1.69) | 0.699 |  |  |
| 5-10 k | 0.83 (0.52-1.32) | 0.428 |  |  |
| Parents work |  |  |  |  |
| Full-time | Ref |  | Ref |  |
| Others | 1.10 (0.68-1.79) | 0.690 | 1.18 (0.70-1.96) | 0.523 |
| Part-time | 0.94 (0.56-1.57) | 0.817 | 1.10 (0.64-1.88) | 0.721 |
| Unemployed | 2.20 (1.11-4.36) | 0.024 | 2.04 (0.96-4.35) | 0.064 |
| Parents health condition |  |  |  |  |
| General | Ref |  |  |  |
| Good | 0.67 (0.36-1.22) | 0.188 |  |  |
| Poor | 1.58 (0.09-26.78) | 0.752 |  |  |
| Parents health insurance |  |  |  |  |
| Basic medical insurance | Ref |  |  |  |
| Commercial insurance | 1.20 (0.66-2.16) | 0.550 |  |  |
| No | 1.46 (0.61-3.49) | 0.392 |  |  |
| Rural cooperative medical insurance | 0.84 (0.52-1.35) | 0.468 |  |  |
| Parents influenza vaccination |  |  |  |  |
| No | Ref |  |  |  |
| Yes | 1.06 (0.42-2.66) | 0.896 |  |  |
| Parents influenza vaccination time |  |  |  |  |
| 0 | Ref |  |  |  |
| 1 | 0.46 (0.05-3.93) | 0.475 |  |  |
| 2 | 2.28 (0.32-16.32) | 0.413 |  |  |
| 3 | 0.91 (0.17-4.75) | 0.912 |  |  |
| 4 | 1.52 (0.25-9.18) | 0.649 |  |  |
| Parents influenza |  |  |  |  |
| No | Ref |  |  |  |
| Yes | 0.64 (0.30-1.38) | 0.253 |  |  |
| Parents friend influenza |  |  |  |  |
| No | Ref |  |  |  |
| Yes | 0.67 (0.43-1.03) | 0.070 |  |  |
| Parents COVID-19 |  |  |  |  |
| No | Ref |  |  |  |
| Yes | 0.82 (0.56-1.20) | 0.305 |  |  |
| Parents friend COVID-19 |  |  |  |  |
| No | Ref |  |  |  |
| Yes | 0.70 (0.48-1.02) | 0.065 |  |  |
| Number of people over 60 years old |  |  |  |  |
| 0 | Ref |  |  |  |
| 1 | 1.22 (0.69-2.15) | 0.497 |  |  |
| ≥ 2 | 0.93 (0.58-1.52) | 0.779 |  |  |
| Number of children |  |  |  |  |
| 1 | Ref |  |  |  |
| 2 | 1.43 (0.95-2.15) | 0.086 |  |  |
| ≥ 3 | 1.17 (0.64-2.10) | 0.595 |  |  |
| K score | 1.25 (1.07-1.46) | 0.006 | 1.19 (1.00-1.43) | 0.052 |
| A score | 1.23 (1.11-1.36) | <0.001 | 1.17 (1.05-1.31) | 0.007 |

OR: odds ratio; CI: confidence intervals; Ref: reference.

K: knowledge; A: attitudes.
Model 1: Crude model.

Model 2: Included variables with statistical differences in model 1.
